# Protein folds and catalytic strategies at the origin of biological CO_2_ fixation

**DOI:** 10.1093/femsec/fiag041

**Published:** 2026-04-22

**Authors:** Natalia Mrnjavac

**Affiliations:** Institute of Molecular Evolution, Faculty of Mathematics and Natural Sciences, Heinrich Heine University Düsseldorf, 40225Düsseldorf,Germany

**Keywords:** acetyl-CoA pathway, chemolithoautotrophs, protein fold evolution, ancient enzymes, biochemical evolution, origin of metabolism

## Abstract

Biological carbon fixation is the basis of all ecosystems. Out of the seven known pathways organisms use to fix CO_2_, the acetyl-CoA pathway is assumed to be the most ancient. Its enzymes bear primordial traits, including carbon–metal bonds and an abundance of transition metal clusters. Ancient traits can also be reflected in structural folds adopted by the enzymes and the catalytic strategies they employ. Here I show that the most common catalytic strategies used to accelerate reactions in the acetyl-CoA pathway are cofactor-aided catalysis, electrostatic interactions and general acid/base catalysis. Enzymes of the acetyl-CoA pathway are replete with Rossman folds, TIM barrel folds, and alpha-beta plaits. These topologies evolved before the divergence of bacteria and archaea, along with five other catalytic folds of the acetyl-CoA pathway. Rossmann folds and TIM barrels likely underwent rapid diversification early in the history of life. In contrast, over half of the folds in the pathway are weakly diversified folds that emerged only once in the history of life, at the origin of biological CO_2_ fixation. Ancient metabolic pathways point to enzymes with conserved structural cores, which uncover topologies and catalytic strategies employed at the onset of metabolism.

## Teaser

The most ancient pathway of carbon fixation, the acetyl-CoA pathway, is a time capsule that uncovers primordial traits in the evolution of life.

## Introduction

All life on Earth is based on carbon, and all ecosystems ultimately rely on biological carbon fixation. The process of converting CO_2_ from the atmosphere to organics that can be further metabolized by cells is carried out by autotrophs that harness sunlight or chemical reactions as energy source. Net primary production by autotrophs on modern Earth amounts to roughly 100 billion tons of CO_2_ yearly, mostly attributable to phototrophs using the Calvin-Benson-Bassham cycle (Field et al. 1998). There are, however, at least six other carbon fixation pathways by which life fixes the most oxidized form of inorganic carbon, CO_2_, into precursors of metabolic food and building blocks (Fuchs 2011, Sánchez-Andrea et al. 2020). The elucidation of CO_2_-fixing pathways spanned over 70 years, starting with the Calvin-Benson-Bassham cycle (Calvin and Benson 1948, Bassham et al. 1950, Bassham et al. 1954, Quayle et al. 1954, Mayaudon et al. 1957) and the reductive acetyl-CoA pathway (Stephenson and Stickland 1933, Wieringa 1939, Barker et al. 1940, Wood and Harris 1952, Diekert and Thauer 1978, Ragsdale and Wood 1985, Fuchs 1986, Wood et al. 1986, Fuchs and Stupperich 1986). Just over a decade after the initial characterization of the Calvin-Benson-Bassham cycle, the reductive citric acid cycle (rTCA) (Evans et al. 1966) was discovered, around the same time the reductive acetyl-CoA pathway was recognized as a distinct CO_2_ fixation pathway (Ljungdahl and Wood 1969). The last described carbon fixation pathway was the reductive glycine pathway, found to support autotrophic growth in 2020 (Sánchez-Andrea et al. 2020), even though its autotrophic potential has been discussed roughly 40 years ago (Fuchs 1986, Wood et al. 1986).

Although the Calvin-Benson-Bassham cycle is the most studied and predominant carbon fixation pathway in terrestrial and marine ecosystems, the reductive acetyl-CoA pathway is of particular interest for several reasons. Organisms employing the acetyl-CoA pathway affect global climate by altering the concentrations of two potent greenhouse gasses, CO_2_ and methane, with methanogens producing ∼10^9^ tons of methane each year (Thauer 1998). Moreover, the pathway is emerging as a promising avenue for the sustainable biotechnological production of biofuels and other chemicals from industrial waste gases, such as syngas, or from greenhouse gases (Zhang et al. 2024). Metabolically engineered, resting acetogen cells in a bioreactor showed promise as “bio-batteries” for H_2_ storage and release (Schwarz et al. 2022).

In addition to its potential as a tool towards a hydrogen economy and carbon neutrality, the acetyl-CoA pathway has been intensively studied in an evolutionary context. In 1970, acetogenic clostridia and methanogens were suggested to be most similar to primordial prokaryotes among modern life forms based on their biochemistry and physiology (Decker et al. 1970). These organisms can typically perform both the reductive acetyl-CoA pathway and the reductive citric acid cycle, but the latter is employed to supply central metabolites, as was probably the case in ancestral life forms. The rTCA is unlikely to be the primordial CO_2_ fixation pathway as only bacteria use it autotrophically (Berg et al. 2010) and, being a cycle, it requires pre-existing organics to run. The acetyl-CoA pathway was proposed to be the ancestral carbon fixation route due to its occurrence in both bacterial and archaeal anaerobes, its ability to accept various C1 substrates, its reliance on metals and coenzymes, and its energetics (Fuchs and Stupperich 1985, Berg et al. 2010, Fuchs 2011). It is the only carbon fixation pathway that can simultaneously conserve energy by generating a chemiosmotic gradient, in which case its end products are methane (in archaea) and acetate (in bacteria) (Fuchs 2011). Phylogenetic reconstructions and sequence analysis have repeatedly traced the acetyl-CoA pathway to the last universal common ancestor (LUCA) (Sousa and Martin 2014, Weiss et al. 2016, Moody et al. 2024). In line with the pathway’s proposed antiquity, carbon isotope evidence for biogenic methane has been found in ∼3.5 billion years old (Ga) hydrothermal precipitates (Ueno 2006 et al. 2006). Isotope measurements from Tashiro and colleagues placed the oldest autotrophs at over 3.95 Ga (Tashiro et al. 2017), although this and other deep time carbon isotope studies are often contested due to challenges in dating different parts of the rock and in differentiating biotic and abiotic formations (Fedo and Whitehouse 2002, Guo et al. 2024). Novel machine learning-based techniques could help to constrain the geological age of the earliest life (Wong et al. 2025).

A strong argument in favor of the acetyl-CoA pathway being not only ancient, but the starting point of metabolism, comes from experiments that reported a non-enzymatic version of the pathway under mild aqueous conditions from CO_2_ and H_2_ (externally provided or internally generated from H_2_O), yielding formate, acetate, pyruvate, and methane over transition metals (Varma et al. 2018, Preiner et al. 2020, Beyazay et al. 2023a, Beyazay et al. 2023b, Song et al. 2024). The remarkable similarity of the geochemical analogue to the enzymatic pathway gave momentum to chemolithoautotrophic theories for the origin of life (Lipmann 1965, Martin and Russell 2007, Martin 2020) that draw on comparative biochemistry and microbial physiology to address the origin and evolution of microbial metabolism at the interface with a geochemically active early Earth environment. The proposed *locus delicti* are Lost City-type hydrothermal vents characterized by mild temperature alkaline effluents (<80 °C) rich in CH_4_ and H_2_ (Kelley et al. 2001, Martin et al. 2008). The latter is produced by serpentinization, a geochemical process that alters ultramafic rock minerals, oxidizing Fe(II) to Fe(III) while reducing H_2_O and simultaneously depositing native metals (Chamberlain et al. 1965, Sleep et al. 2004, Lawley et al. 2020, Schwander et al. 2023). It is suggested that serpentinizing vents could thus provide H_2_ with highly negative reduction potentials (Boyd et al. 2020) and transition metal minerals which are sufficient to drive prebiotic CO_2_ fixation (Varma et al. 2018, Preiner et al. 2020, Beyazay et al. 2023a, Beyazay et al. 2023b, Song et al. 2024).

Under a chemolithoautotrophic origins framework, how did mineral surface-promoted prebiotic reactions evolve into *bona fide* cells bolstering intricate metabolic networks that sustain steady state concentrations of metabolites, all while maintaining redox balance and conserving energy? Catalysis is key. Transition metals promoting early prebiotic chemistry would at some point need to be replaced by enzymes and enzyme-cofactor complexes, with their superior rate enhancement (Wolfenden 2011) and specificity (Jensen 1976), in order for metabolism to break free from surfaces and increase in complexity. The prokaryotic cellular machinery evolved largely around protein synthesis: an average bacterial cell is roughly 50% protein by dry weight (Stouthamer 1973), most of bacterial DNA (about 90%) encodes protein-coding genes (Lybecker et al. 2014), and protein synthesis takes up 75% of the total ATP budget of the cell (Mrnjavac and Martin 2025a). However, not all enzymes evolved simultaneously, and the acetyl-CoA pathway that provided energy and organics at the onset of metabolism likely harbors some of the most ancient enzymes, dating to the earliest stages of metabolic evolution.

Ancient enzymes bear ancient traits (Eck and Dayhoff 1966). Some of these traits are relics of the kind of primordial transition metal-driven chemistry that kickstarted life: enzymes of the acetyl-CoA pathway harbour carbon–metal bonds (Martin 2019) and are enriched in catalytic metal centers that contain Fe, Ni, Co, Mo or W (Nicolet et al. 1999, Hiromoto et al. 2009, Kung et al. 2012, Wagner et al. 2017, Biester et al. 2024, Yin et al. 2025, Basak et al. 2025, Ohmer et al. 2025). Four billion years later, these enzymes have preserved evidence for the ancestral tether between biochemistry and geochemistry. In addition, some aspects of ancient enzymes might uncover inventions that allowed biochemistry to gradually separate from surface chemistry: the folds and domains they employ and their specific catalytic strategies.

Protein structures are more conserved than sequences (Chothia and Lesk 1986) because directly selected for function, and can therefore trace deeper in time than sequences can, a property increasingly employed in evolutionary studies (Mrnjavac et al. 2025b, Modjewski et al. 2025, Garg and Hochberg 2025, Moi et al. 2025). Here, enzymes of the acetyl-CoA pathway were used as time capsules that preserve ancient biochemical traits, with the aim to investigate protein folds and catalytic strategies that had to be in place before the divergence of bacteria and archaea. Ancestral metabolic pathways such as the acetyl-CoA pathway point to ancient proteins, and therefore to ancient structural and catalytic inventions.

## Materials and methods

A list of enzymes of the acetyl-CoA pathway with E.C. numbers was compiled from the Kyoto Encyclopedia of Genes and Genomes (KEGG) (Kanehisa and Goto 2000) and the literature (Ferry 1999, Shima et al. 2002, Ragsdale and Pierce 2008, Thauer et al. 2010) (Supplementary Table 1). The Mechanism and Catalytic Site Atlas (M-CSA) (Ribeiro et al. 2018) was queried by E.C. number, and the roles of catalytic residues and CATH superfamily assignments were retrieved for 9 enzymes of the pathway. Catalytic residues of methyl-coenzyme M reductase (MCR) and formate dehydrogenase were manually curated because residue annotation from M-CSA was not supported by current experimental evidence (Supplementary Table 1). Residues that participate only in ligand binding are not included in M-CSA. CO dehydrogenase (CODH), acetyl-CoA synthase (ACS), phosphotransacetylase (Pta), and methyltetrahydrofolate:corrinoid iron-sulfur protein (methyl-H_4_F:CoFeSP) methyltransferase (MeTr) did not have an M-CSA entry, so catalytic residues were manually annotated based on the literature (listed in the legend of Table 1). When catalytic residues were already assigned for the bacterial enzyme, the archaeal counterpart was not hand-annotated. In-house Python scripts were used to retrieve and curate the data.

For the nine enzymes of the pathway represented in M-CSA, both the CATH superfamily for the catalytic domain (indicated in M-CSA) and the CATH superfamilies of the remaining domains were retrieved (Supplementary Table 1). CATH assignments for enzymes not represented in M-CSA were obtained from representative PDB structures, with no distinction between catalytic and non-catalytic domains. Statistical significance of the frequencies of fold superfamilies relative to the CATH database and of catalytic roles relative to the M-CSA database were tested with the hypergeometric test from the SciPy Python package (Virtanen et al. 2020) (p-value threshold of 0.05), applying the Benjamini-Hochberg correction (false discovery level of 0.05) (Supplementary Table 1). In-house Python scripts were used to determine amino acid role counts in the complete M-CSA database.

Bacterial and archaeal protein structures for Fig. 3 were retrieved from the Protein Data Bank (PDB) (Armstrong et al. 2020). The structure of methyltetrahydromethanopterin:coenzyme M methyltransferase (Mtr) is a combination of two experimental structures and an AlphaFold 3 (Abramson et al. 2024) model, generated similarly as in Aziz et al. 2024, and as described in Mrnjavac et al. 2025b. When possible, CATH classifications were used to determine the position of Rossmann folds, TIM barrels, and alpha-beta plaits. For enzymes lacking a PDB structure with CATH-classified domains, fold assignment for Fig. 3 was based on homologous enzymes and the literature, with occasional help from PFAM domain classifications. Namely, fold assignment for PDB ID 9C0T was based on structural alignments with PDB ID 3I01, PFAM domains and Biester et al. 2024; fold assignment for PDB ID 6X5K was based on the structure PDB ID 3I01 from the same organism; fold assignment for PDB ID 5ODC was based on Wagner et al. 2017; fold assignment for PDB ID 6CIN and PDB ID 9BT4 was based on structural alignments with PDB ID 2C3M and Cossu et al. 2024; fold assignment for PDB ID 5T5M was based on Wagner et al. 2016a and PFAM domains; fold assignment for Mtr was based on Aziz et al. 2024 and PFAM domains.

## Results and discussion

### Ancient enzymes used the same catalytic strategies as modern ones

Thermodynamic barriers to metabolic reactions in modern cells are overcome by employing chemical energy carriers such as ATP and NAD(P)H or reduced ferredoxin (Thauer et al. 1977, Herrmann et al. 2008, Müller et al. 2018). In contrast, overcoming kinetic barriers requires the costly production of highly specialized biocatalysts—enzymes. How did ancient enzymes catalyze the first metabolic reactions so as to replace their forerunners, inorganic metal catalysts? The acetyl-CoA pathway might hold clues. Although about half of modern enzymatic reactions require cofactors (Fischer et al. 2010), all enzymes of the acetyl-CoA pathway rely on them, including metal clusters and a variety of organic coenzymes. With such a strong dependence on cofactor-mediated catalysis, what role did amino acid catalysis play in ancient enzymes?

Before answering this question, a central premise of the present study needs to be spelled out clearly: We assume that the reaction mechanism of a given enzyme in this sample has not changed since its origin. That is, the likelihood of existing catalytic strategies for essential functions significantly changing over time is taken here as negligibly low, as it would require a reinvention of the enzyme’s active site, interrupting continuity of its function. Active site tampering would be possible only if the activity could be efficiently rescued by another enzyme with broad specificity, or if the change occurs in duplicated genes, resulting in functional diversification within an enzyme family (Jensen 1976). Neither of these, however, include reverting to the original function. As a result, residues responsible for catalysis in enzymes that retain the same function over time are generally conserved, and therefore appear highly recalcitrant to change. It is thus reasonably safe to assume that catalytic strategies employed at the active site of early enzymes are the same as those employed today. The same mechanism and active site architecture could also originate multiple times independently through convergent evolution, as in the case of glycosyl hydrolases (Davidi et al. 2018).

**Figure 1:**
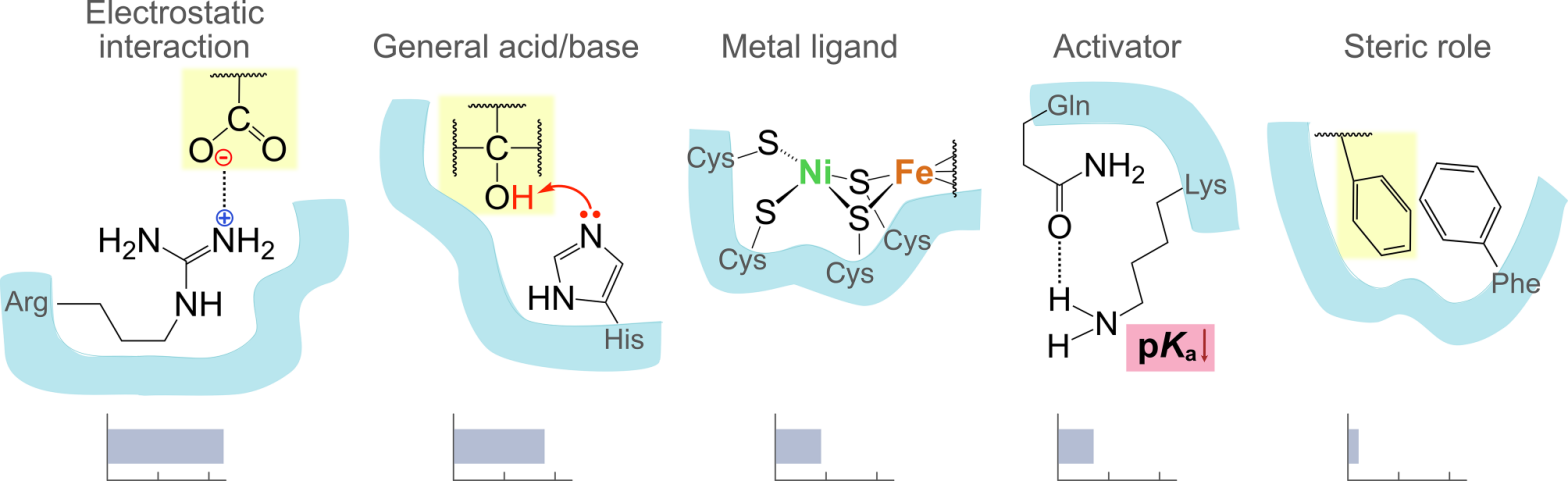
**General functions of catalytic amino acids in enzymes of the acetyl-CoA pathway**. Bars show the occurrence of each general function (ticks are set at 10 and 20 occurrences), along with a schematic example. The substrate or reaction intermediate is highlighted in yellow. The active site pocket is shown as a light blue surface. A stabilizing salt bridge between a catalytic arginine and the charged intermediate is shown as an example of electrostatic interaction. Residues involved only in ligand binding are not considered catalytic, but those that coordinate a catalytic metal are regarded as catalytic residues. Glutamine acts as an activator in the example because it decreases the side chain p*K*_a_ of the nearby catalytic lysine by interacting with its deprotonated form. Phenylalanine fulfils a steric catalytic function by positioning the reacting species to induce reaction. A detailed description of catalytic roles can be found in the documentation of the M-CSA database.

To gain insight into the function of catalytic amino acid residues in early enzymes, the general functions and specific roles of catalytic residues in acetyl-CoA pathway enzymes were retrieved from the M-CSA database or manually annotated. Out of the enzymes listed in Supplementary Table 1, nine had an M-CSA entry (Table 1). These include the complete bacterial methyl branch, acetate kinase, pyruvate synthase (PFOR), and the archaeal methane synthesis enzyme MCR. Catalytic residues for MCR and formate dehydrogenase were additionally manually curated (marked with * in Table 1), and residues for CODH, ACS, phosphotransacetylase, and MeTr were annotated from the literature (marked with ** in Table 1). General catalytic functions are shown in Fig. 1 and listed in Table 1, while specific roles are listed in Supplementary Table 1. In spite of the small sample size, the most common catalytic residues align with the most frequent ones in the M-CSA database: positively and negatively charged amino acids and histidine. Lysine is employed in catalysis by seven enzymes of the acetyl-CoA pathway, histidine and arginine by six enzymes each, aspartate by five enzymes, and serine and glutamate by four different enzymes each (Table 1). When specific catalytic roles in the acetyl-CoA pathway are compared to all entries in the M-CSA database, enzymes of the pathway emerge as enriched in amino acids mediating proton relay in catalysis (corrected p-value 1.04×10^−3^), while other roles are in line with their background frequencies (Supplementary Table 1). The most common general catalytic strategies used by residues in the sample are electrostatic interaction and general acid/base catalysis (Fig. 1), which aligns with expectations for enzymes in general (Fried and Boxer 2017, Ribeiro et al. 2020). Even though one instance of covalent catalysis mediated by selenocysteine was identified in the database for formate dehydrogenase, studies show selenocysteine likely participates only by metal binding, or in concert with general acid/base catalysis, but does not form a covalent intermediate (Niks and Hille 2019). Glycine-mediated one-electron shuttle was listed in the M-CSA database for MCR, however recent experimental evidence points to a methyl radical-based mechanism without amino acid involvement in single electron transfer (Wongnate et al. 2016, Thauer 2019, Ohmer et al. 2025). These uncorroborated annotations were excluded from the analysis in favor of manually curated catalytic roles.

**Table 1:** General roles of catalytic residues in enzymes of the acetyl-CoA pathway.

*Formate dehydrogenase* *		
	Arg, Lys	electrostatic interaction
	Sec	metal ligand, proton shuttle (general acid/base)
	His	proton shuttle (general acid/base), electrostatic interaction
*10-formyl-H_4_F synthetase*		
	Lys, Arg, Ala	electrostatic interaction
	Trp	activator
	Thr	steric role
	Phe	activator, electrostatic interaction
*5,10-methenyl-H_4_F cyclohydrolase*		
	Gln, Ser	activator
	Lys	proton shuttle (general acid/base)
*5,10-methylene-H_4_F dehydrogenase*		
	Lys, Gln	activator
	Asp	electrostatic interaction
*5,10-methylene-H_4_F reductase*		
	His, Ser, Glu	proton shuttle (general acid/base)
	Phe	steric role
	Asp	electrostatic interaction
*Acetate kinase*		
	Asn, Glu	metal ligand
	Arg, His	electrostatic interaction
*Ferredoxin:H_2_ hydrogenase*		
	Glu, Ser, Lys	proton shuttle (general acid/base)
	Cys	proton shuttle (general acid/base), metal ligand
*Pyruvate:ferredoxin oxidoreductase*		
	Glu	proton shuttle (general acid/base)
	Asn, Arg, Thr	electrostatic interaction
Methyl-coenzyme M reductase *		
	Gln	metal ligand, electrostatic interaction
	Arg	electrostatic interaction
	Tyr	electrostatic interaction
	Lys	electrostatic interaction
	His	electrostatic interaction
*Phosphotransacetylase* **		
	Asp	proton shuttle (general acid/base)
	Ser	electrostatic interaction
	Arg	activator
CODH **		
	Cys	metal ligand
	His	metal ligand, electrostatic interaction, proton shuttle (general acid/base)
	Lys	electrostatic interaction, proton shuttle (general acid/base)
	Asp, Trp	proton shuttle (general acid/base)
*ACS* **		
	Cys, Gly	metal ligand
	His	proton shuttle (general acid/base)
*Methyl-H_4_F:CoFeSP methyltransferase* **		
	Asn	electrostatic interaction
	Asp	proton shuttle (general acid/base)

For enzymes with an entry in the M-CSA database, mechanism identifiers can be found in Supplementary Table 1. In the case of formate dehydrogenase and MCR (marked with *), some residue roles in the database are not supported by experimental evidence, so residue roles were manually curated (Niks and Hille 2019, Wongnate et al. 2016, Ohmer et al. 2025). For acetyl-CoA pathway enzymes not represented in the M-CSA database (marked with **), catalytic residue functions were manually annotated based on the literature (Dobbek et al. 2001, Lawrence et al. 2006, Doukov et al. 2007, Volbeda et al. 2009, Can et al. 2014, Chen and Siegbahn 2020, Newman-Stonebraker et al. 2024, Basak et al. 2025, Yin et al 2025).

Modern enzymatic catalysis is carried out by a small number of residues that employ a limited number of catalytic strategies (Ribeiro et al. 2020). In spite of prosthetic groups and coenzymes performing much of the chemistry in the acetyl-CoA pathway, a variety of general amino acid catalytic strategies was identified (Table 1, Fig. 1). This points to the possibility of many amino acid-based catalytic mechanisms evolving at the origin of enzymes. Even functions that are facile for metals but demanding for enzymes, such as radical chemistry, trace to ancient enzymes (MCR is a radical enzyme) (Mrnjavac et al. 2024). Some specific residue roles in catalysis do not occur in the current sample, such as specific types of activation, electrostatic destabilization, or covalent catalysis. This can be a result of some catalytic strategies being very rare in general (according to M-CSA) and the current sample of ancient enzymes being small, but could also point to some modes of catalysis emerging after the origin of enzymes of the acetyl-CoA pathway. Some catalytic strategies are difficult to assign, such as the effects of entropy (Åqvist et al. 2017), desolvation (Rucker and Byers 2000), electric fields (Fried and Boxer 2017), etc. Increasing the sample of ancient enzymes will likely expand the list of ancient catalytic mechanisms, and perhaps even uncover an order in the evolution of enzymatic catalytic strategies.

It is unclear whether and to which extent the mechanisms of primordial non-enzymatic reactions resemble those employed by enzymes. Besides transition metals, protometabolic catalysis could have been aided by concentration mechanisms such as thermophoresis (Matreux et al. 2024) or by microdroplet chemistry, which can include electric field effects and could play a part in overcoming not only kinetic, but thermodynamic barriers in challenging protometabolic reactions (Nam et al. 2018, Ju et al. 2023, Piejko et al. 2025, Wang et al. 2025, Mohajer et al. 2025).

### The acetyl-CoA pathway preserves ancient folds

Decades of structural studies have resulted in a number of experimental structures for enzymes of the acetyl-CoA pathway (Ermler et al. 1997, Kung et al. 2012, Wagner et al. 2016a, Wagner et al. 2017, Shima et al. 2020, Biester et al. 2024, Yin et al. 2025, Ohmer et al. 2025, Basak et al. 2025). Perhaps unexpectedly, some of the enzymes have been shown to form large complexes in modern cells, both in methanogens (Wagner et al. 2017, Watanabe et al. 2021, Biester et al. 2024) and in acetogens (Dietrich et al. 2022). Molecular motion and conformational changes have been described in enzymes such as CoFeSP/MeTr and CODH/ACS (Kung et al. 2012, Yin et al. 2025). If the enzymes of the acetyl-CoA pathway are ancient, are these traits also ancient? The most parsimonious explanation is that the enzymes in question were simpler at the time of origin, but over 4 billion years evolved features that conferred selective advantage by increasing efficiency or stability.

**Figure 2:**
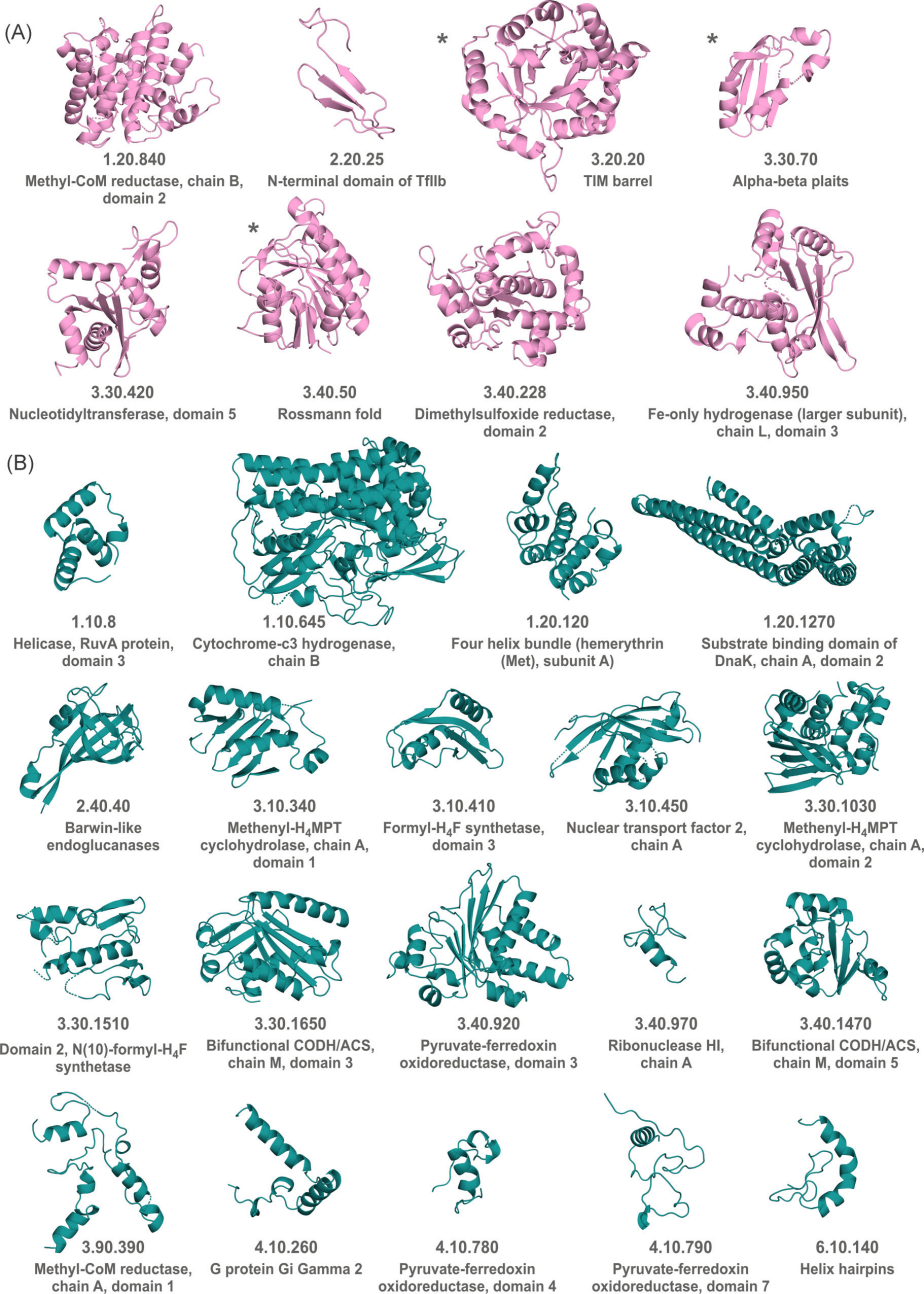
**Structural folds of enzymes in the acetyl-CoA pathway**. Structures belong to example domains listed in CATH for the respective CATH topologies. (A) Pink folds are adopted by catalytic domains of enzymes of the acetyl-CoA pathway as assigned by M-CSA. (B) Green folds are adopted by either non-catalytic domains, or domains which could not be identified as catalytic or non-catalytic (for enzymes without an M-CSA entry). Folds marked with * in (A) (Rossmann, alpha-beta plaits and TIM barrels) also occur in non-catalytic or unassigned domains, but are omitted from (B) for reasons of space. The only folds employed more than once in the acetyl-CoA pathway are the Rossmann fold (16 occurrences), alpha-beta plaits (five occurrences), TIM barrels (four occurrences) and Barwin-like endoglucanases (two occurrences).

The fundamental structural unit of early enzymes would include the catalytic core shaping the active site. By the same reasoning applied to catalytic mechanisms, the catalytic core is unlikely to have changed significantly over time, as it dictates the fundamental enzymatic function. This makes catalytic domains a proxy for ancient structural signatures. Drawing on this principle, the folds of acetyl-CoA pathway enzymes (Supplementary Table 1) were determined based on the third classification level in the CATH database (Orengo et al. 1997) corresponding to topology/fold in order to obtain a list of ancient fold candidates. The folds (CATH topologies) of acetyl-CoA pathway enzymes that have a CATH entry are shown in Fig. 2. They span all five structural classes in CATH (mainly alpha, mainly beta, alpha beta, few secondary structures, and special), with most belonging to the alpha beta class (class 3), the most common fold class in the database. The most frequent architecture is a 3-layer sandwich (CATH 3.40), adopted by 21 out of the 50 classified folds. Folds adopted by catalytic domains were identified for roughly half of the enzymes of the acetyl-CoA pathway, as these have an entry in the M-CSA database (Table 1). The folds adopted by catalytic domains in this sample are: the MCR chain B domain 2 fold, the N-terminal domain of TFIIb fold, the TIM barrel fold, the alpha-beta plait fold, the nuleotidyltransferase domain 5 fold, the Rossmann fold, the dimethylsulfoxide reductase domain 2 fold, and the [Fe] hydrogenase (larger subunit) chain L domain 3 fold (Fig. 2A).

Based on the antiquity of the pathway, the catalytic domain folds in Fig. 2A were in place before or during the split between archaea and bacteria. This holds true also for some of the folds in Fig. 2B, as they belong to catalytic domains that could not be identified from the databases used. The remaining folds in Fig. 2B are adopted by domains with other, non-catalytic functions. Some of these are also likely ancient, in which case they would be added to the core functional domain before the origin of cells. For example, it is conceivable that early enzymes were accepting electrons directly from metal surfaces, as has been shown experimentally for ferredoxin (Brabender et al. 2024). However, for metabolism to become soluble, domains that accommodate soluble electron donors and mediate electron transfer would have to be added to catalytic domains.

**Figure 3:**
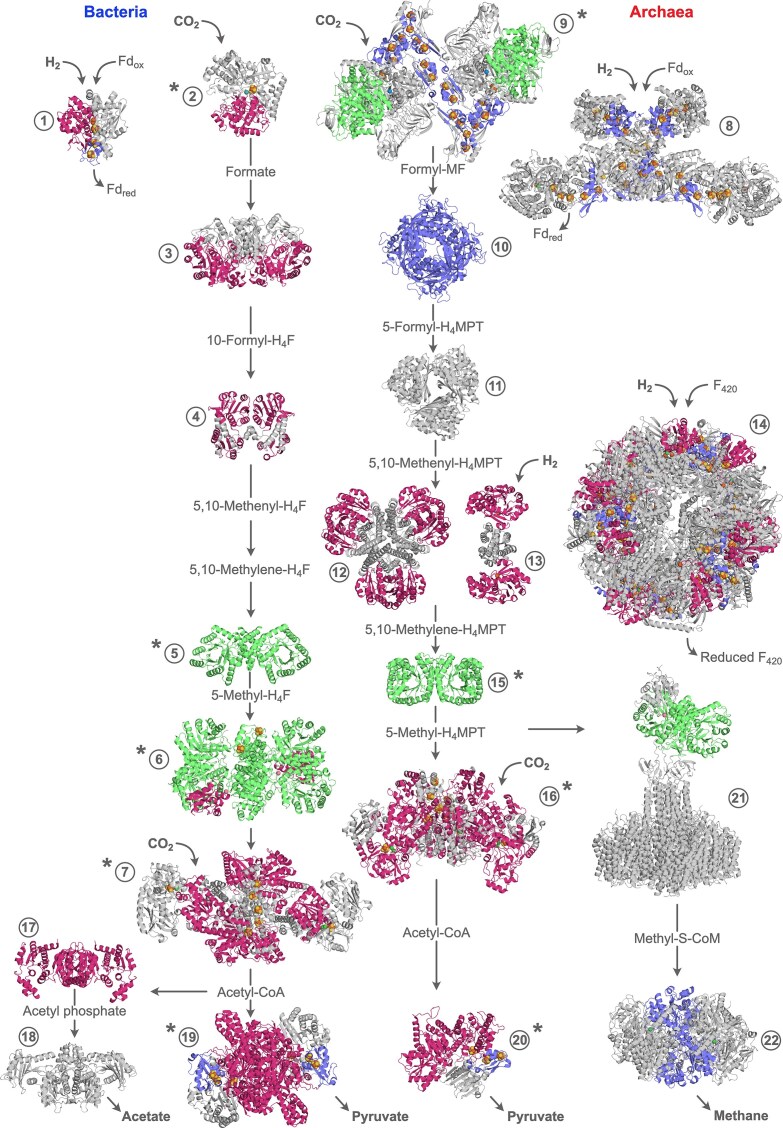
**Rossmann folds, TIM barrels and alpha-beta plaits in enzymes of the acetyl-CoA pathway**. PDB structures of enzymes of the acetyl-CoA pathway from H_2_ and CO_2_ to pyruvate, acetate and methane in bacteria (left) and archaea (right) are shown. The main reaction substrates and products are indicated. H_2_ and CO_2_ are highlighted where they enter the pathway. Rossmann folds are shown in pink, TIM barrels in green and alpha-beta plaits in blue. No CATH entry was available for enzymes 8 and 21, and most domains were unassigned for enzyme 9, so Rossmann folds, TIM barrels and alpha-beta plaits were identified from the literature. They are highlighted here for the sake of completeness, although not counted in the CATH database fold analysis. Transition metals and metal clusters are shown as spheres. When a transition metal is part of an organic cofactor, such as in the corrinoid cofactor of CoFeSP or in coenzyme F_430_, only the metal atom is shown. The number of subunits and domain composition can vary between organisms. Enzymes that exhibit sequence and/or structural similarity between archaea and bacteria are marked with * (based on Wagner et al. 2016b, Sousa and Martin 2014, Cossu et al. 2024, Modjewski et al. 2025). The enzymes of the acetyl-CoA pathway to acetyl-CoA in bacteria and their PDB structures are: enzyme 1: ferredoxin:H_2_ hydrogenase (Hyd, [FeFe] hydrogenase, PDB: 1HFE (Nicolet et al. 1999)); enzyme 2: formate dehydrogenase (Fdh, PDB: 1AA6 (Boyington et al. 1997)); enzyme 3: 10-formyltetrahydrofolate synthetase (Fhs, formate–tetrahydrofolate ligase, PDB: 1EG7 (Radfar et al. 2000)); enzyme 4: 5,10-methylenetetrahydrofolate dehydrogenase/cyclohydrolase (FolD, PDB: 1B0A (Shen et al. 1999)); enzyme 5: 5,10-methylenetetrahydrofolate reductase (MetF, PDB: 1ZP3 (Pejchal et al. 2005)); enzyme 6: CoFeSP/methyltetrahydrofolate:CoFeSP methyltransferase (CoFeSP/MeTr complex, PDB: 4DJE (Kung et al. 2012)); enzyme 7: carbon monoxide dehydrogenase/acetyl-CoA synthase (CODH/ACS complex, PDB: 6X5K (Cohen et al. 2020)). The enzymes of the acetyl-CoA pathway to acetyl-CoA in archaea are: enzyme 8: heterodisulfide reductase/[NiFe] hydrogenase (Hdr-Mvh complex, PDB: 5ODC (Wagner et al. 2017)); enzyme 9: formylmethanofuran dehydrogenase (Fwd/Fmd, PDB: 5T5M (Wagner et al. 2016a)); enzyme 10: formylmethanofuran:tetrahydromethanopterin formyltransferase (Ftr, PDB: 2FHK (Acharya et al. 2006)); enzyme 11: 5,10-methenyltetrahydromethanopterin cyclohydrolase (Mch, PDB: 1QLM (Grabarse et al. 1999)); enzyme 12: F_420_-dependent 5,10-methylenetetrahydromethanopterin dehydrogenase (Mtd, PDB: 1QV9 (Hagemeier et al. 2003)); enzyme 13: H_2_-forming 5,10-methylenetetrahydromethanopterin dehydrogenase (Hmd, [Fe] hydrogenase, PDB: 3F47 (Hiromoto et al. 2009)); enzyme 14: F_420_-reducing [NiFe] hydrogenase (Frh, PDB: 4CI0 (Allegretti et al. 2014)); enzyme 15: F_420_-dependent 5,10-methylenetetrahydromethanopterin reductase (Mer, PDB: 1F07 (Shima et al. 2000)); enzyme 16: acetyl-CoA decarbonylase/synthase (ACDS complex, PDB: 9C0T (Biester et al. 2024)). In the latter, the CoFeSP subunit is missing from the experimental structure, and therefore not shown. The enzymes of acetogenesis are: enzyme 17: phosphotransacetylase (Pta, phosphate acetyltransferase, PDB: 1QZT (Iyer et al. 2004)); enzyme 18: acetate kinase (Ack, PDB: 1G99 (Buss et al. 2001)). The enzyme catalyzing pyruvate synthesis is pyruvate:ferredoxin oxidoreductase (Pfor, pyruvate synthase). The bacterial pyruvate synthase is enzyme 19 (PDB: 6CIN (Chen et al. 2018)) and the archaeal pyruvate synthase is enzyme 20 (PDB: 9BT4 (Cossu et al. 2024)). The enzymes of methanogenesis are: enzyme 21: 5-methyltetrahydromethanopterin:coenzyme M methyltransferase (Mtr, PDB: 8Q3V (Aziz et al. 2024), 5LAA (Wagner et al. 2016b)); enzyme 22: methyl-coenzyme M reductase (Mcr, PDB: 1MRO (Ermler et al. 1997)). Abbreviations: CoFeSP: corrinoid iron-sulfur protein; Fd: ferredoxin; ox: oxidized; red: reduced; H_4_F: tetrahydrofolate; H_4_MPT: tetrahydromethanopterin.

Acetyl-CoA pathway enzymes likely emerged over a period spanning from before LUCA until after the divergence of the two prokaryotic domains, resulting in a striking homology pattern (Fig. 3). CODH/ACS, CoFeSP and PFOR are homologous between bacteria and archaea (Sousa and Martin 2014, Cossu et al. 2024), suggesting that LUCA could enzymatically catalyze the synthesis of C2 (Weiss et al. 2016, Moody et al. 2024) and maybe even C3 compounds from C1 units (Fig. 3). However, the synthesis of reduced C1 compounds (the methyl branch) is largely non-homologous between the two domains of life, with two instances of measurable structural similarity: (i) bacterial formate dehydrogenase subunit FdhA and archaeal formylmethanofuran dehydrogenase subunits FwdBD (Wagner et al. 2016b) share the Barwin-like endoglucanase fold and other parts of the topology (not explicitly listed here because the other folds of formylmethanofuran dehydrogenase do not have a CATH classification), and (ii) methylenetetrahydrofolate reductase and methylenetetrahydromethanopterin reductase (Modjewski et al. 2025) both consist of TIM barrels, albeit belonging to different CATH homologous superfamilies (Fig. 3). Other enzymes of the methyl branch adopt different topologies in bacteria and archaea (Supplementary Table 1), suggesting they originated independently after LUCA, which would have still relied on abiotic sources of reduced C1 compounds (Weiss et al. 2016).

The current sample includes not only enzymes of carbon fixation to acetyl-CoA and pyruvate, which would be required at the earliest stage of enzymatic metabolism, but also enzymes of acetogenesis and methanogenesis. Are these processes equally ancestral as the total synthesis of acetyl-CoA? Modern chemolithoautotrophic theories for the origin of metabolism not only posit that primordial CO_2_ fixation was promoted by environmental metals (Beyazay et al. 2023a, Beyazay et al. 2023b), Varma et al. 2018, Preiner et al. 2020, Song et al. 2024), but also that primordial energy conservation was reliant on environmental chemistry. A geochemical version of the AMP-dependent phosphite dehydrogenase reaction (Mao et al. 2023) reveals that substrate-level phosphorylation (SLP)-like reactions could proceed on metals with phosphite as phosphoryl-donor (Mrnjavac et al. 2025b). In addition, before hydrogenases and flavin-based electron bifurcation, metal surfaces could promote ferredoxin reduction with H_2_ (Brabender et al. 2024). At a later stage, the ATP synthase, shown to function in fatty acid vesicles (Yu et al. 2025), could perhaps harness environmental proton gradients such as those naturally occurring at the vent–ocean interface (Martin and Russell 2007). In order for cells to emerge, reliance on environmental surface chemistry had to be abandoned for both carbon fixation and energy conservation. This was made possible by enzymes. The independent origin of enzymes of the methyl branch in bacteria and archaea (Sousa and Martin 2014, Modjewski et al. 2025) rendered carbon fixation fully soluble. Meanwhile, the emergence of enzymes for acetogenesis in bacteria and methanogenesis in archaea (Fig. 3) allowed for energy conservation to become independent of geochemical ion gradients and metal surfaces.

### Rossman folds, TIM barrels and alpha-beta plaits make up 50% of folds in the acetyl-CoA pathway

Sequence space is vast, but structural diversity is limited to a relatively small number of protein folds: about 1400 topologies (folds) are classified in the CATH database. Folds are structural, not evolutionary categories. Each topology (fold) encompasses a number of CATH homologous superfamilies, given that folds can also arise by convergence, or simply lose any detectable homology signal. It can be challenging to differentiate between these two cases, like in the case of bacterial and archaeal methylenetetrahydropterin reductases from the acetyl-CoA pathway, which adopt a TIM barrel fold but display no primary sequence homology and differ in the nature of the electron-carrying cofactor (Gehl et al. 2024) (Fig. 3).

Studies in recent years have identified instances of possible common ancestry among and within several groups of modern folds (Longo et al. 2020, Alvarez-Carreño et al. 2022, Yagi and Tagami 2024). For example, although the present sample contains 16 Rossmann fold occurrences that include 13 different homologous superfamilies, one study has proposed a single pre-LUCA ancestor of Rossmann folds (Laurino et al. 2016). Multiple homologous superfamilies with a Rossmann fold (CATH 3.40.50, 13 superfamilies) and TIM barrel fold (CATH 3.20.20, three superfamilies) occur in the sample, and are enriched relative to the CATH database (for statistics see Supplementary Table 1), supporting the notion of repeated recruitment and early evolutionary diversification of these folds. Rossmann folds and alpha-beta plaits (CATH 3.30.70, three superfamilies) carry out both catalytic and non-catalytic functions in the present sample (Supplementary Table 1), pointing to early functional divergence. This is in line with a previously proposed early and rapid fold diversification period before LUCA (Yagi and Tagami 2024). Another example of likely common ancestry in modern folds are the OB and SH3 folds. They are widely represented in ribosomal proteins and in other components of the translation system and regarded as some of the most ancient folds overall (Alvarez-Carreño et al. 2021, Alvarez-Carreño et al. 2022, Yagi and Tagami 2024), but neither is represented among acetyl-CoA pathway enzymes. Some folds found in ribosomal proteins can nonetheless be identified in the acetyl-CoA pathway, such as the ribonuclease HI fold (CATH 3.40.970) or alpha-beta plaits (CATH 3.30.70).

Besides being enriched in homologous superfamilies of the Rossman and TIM barrel folds, the acetyl-CoA pathway includes a number of weakly diversified folds. The occurrence of even one homologous superfamily from these folds in the sample would in principle represent enrichment, as the folds include one to five superfamilies overall, but interpretation is challenging due to the small sample size. These folds manifest limited diversification over time (they include a small number of homologous superfamilies). Three such folds in the sample originated a few times in the history of life, but most of them (14 folds) seem to have emerged only once (Supplementary Table 1). Folds that originated only once in four billion years, at the origin of enzymatic metabolism, and did not significantly diversify, make up 28% of fold occurrences in the acetyl-CoA pathway, but over half (52%) of identified folds are in this category. These are: the MCR chain B domain 2 fold, the MCR chain A domain 1 fold, the cytochrome-c3 hydrogenase chain B fold, the dimethylsulfoxide reductase domain 2 fold, the Fe-only hydrogenase (larger subunit) chain L domain 3 fold, the folds from bifunctional CODH/ACS chain M domains 3 and 5, the folds of PFOR domains 3, 4, and 7, the N(10)-formyl-H_4_F synthetase domain 2 fold, the folds of methenyl-H_4_MPT cyclohydrolase chain A domains 1 and 2, and the formyl-H_4_F synthetase domain 3 fold.

Out of the 27 different folds adopted by enzymes of the acetyl-CoA pathway with a CATH entry (Fig. 2), only Rossmann folds, TIM barrels, alpha-beta plaits (which include the ferredoxin fold), and Barwin-like endoglucanase folds occur more than once. As much as half (25/50) of acetyl-CoA pathway fold occurrences are classified as either Rossmann folds (32%), TIM barrels (8%), or alpha-beta plaits (10%) (marked in Fig. 3). As an extreme case, the CoFeSP/Metr complex in acetogens is solely made up of TIM barrels and Rossmann folds (Fig. 3). The three most commonly occurring folds in the acetyl-CoA pathway have been previously identified as the most versatile in binding chemically different organic and organometallic cofactors (Sanchez-Rocha et al. 2024). Their plasticity in cofactor binding might have been advantageous in the early stages of enzyme evolution and a reason for their prevalence.

Previous studies reported that Rossmann proteins make up roughly 20% of the PDB, but almost 40% of metabolic enzymes, with enrichment in ancient pathways such as the acetyl-CoA pathway (Medvedev et al. 2019). In line with this, 13 out of the 22 enzymes of the acetyl-CoA pathway reported here (59%) are Rossmann proteins (Fig. 3). TIM barrel proteins make up about 10% of all proteins (Farber et al. 1993), but almost 23% (5 out of 22) of acetyl-CoA pathway enzymes. Rossmann folds, TIM barrels, and alpha-beta plaits emerge as fundamental in the earliest stages of metabolic evolution, in line with a previous study that identified the ferredoxin fold (which adopts an alpha-beta plait topology) and Rossmann-like folds as central to the origin of metabolism (Raanan et al. 2020). They were, however, complemented by specialized ancient weakly diversified folds that emerged only once or a few times in the history of life.

### Limitations of the study

The acetyl-CoA pathway harbours several enzymatic alternatives for the same reactions. Hmd, the [Fe] hydrogenase, is shown as an alternative to Mtd in Fig. 3. Its direct use of H_2_, bypassing organic electron carriers, and the iron-guanylylpyridinol (FeGP) cofactor it employs suggest it might be the ancient alternative (Mrnjavac et al. 2023). Similarly, a hydrogen-dependent CO_2_ reductase (HDCR) has been discovered that reduces CO_2_ to formate with H_2_, with the electrons being transferred by iron-sulfur clusters between active sites, circumventing the need for soluble electron carriers (Schuchmann and Müller 2013, Dietrich et al. 2022). The enzyme was not included because it was not present in CATH nor M-CSA. Some variants of enzyme complexes were omitted for simplicity, for example the Mtd-Hmd system (Afting et al. 1998), in conjunction with F_420_-dependent electron-donating proteins in complex with Hdr, provides electrons for methanogenesis under nickel limitation (Nomura et al. 2025). There are also examples where Hdr complexes not only with a hydrogenase or formate dehydrogenase, but also directly with formylmethanofuran dehydrogenase to which it provides low potential electrons for CO_2_ reduction through a polyferredoxin subunit (Watanabe et al. 2021, Nomura et al. 2024).

The present study did not encompass all folds and all enzyme mechanisms of acetyl-CoA pathway enzymes (Supplementary Table 1) due to database incompleteness, so it is intended to be illustrative rather than exhaustive. Catalytic folds were assigned from the M-CSA database, resulting in a number of enzymes with uncategorized folds, as they did not have an M-CSA entry. Overall fold occurrence in central metabolism would be a natural point of comparison to put the results in context, but reports have so far been limited to a small number of folds. Therefore, only the occurrence of Rossmann and TIM barrel proteins in the acetyl-CoA pathway is discussed in the context of their overall prevalence in metabolism.

Catalytic residue roles of several enzymes not represented in the M-CSA database or with uncorroborated annotations were manually curated, so the assignment criteria may differ from those used by database curators. Moreover, the mechanistic details of some enzymes of the acetyl-CoA pathway are still unresolved, such as CODH/ACS (Cohen et al. 2020, Biester et al. 2022, Newman-Stonebraker et al. 2024, Yin et al. 2025), formate dehydrogenase (Niks and Hille 2019) or MCR (Wongnate et al. 2016, Ohmer et al. 2025). The growth and expansion of databases such as CATH and M-CSA, combined with data from different sources, and further elucidation of enzyme mechanisms will enable increasingly comprehensive studies.

## Conclusion

The assumption underlying this work is that an ancient metabolic pathway roots its enzymes deep. The enzymes, in turn, inform on ancient structural topologies, primordial enzyme mechanisms, and early catalytic residue roles. Half of the fold occurrences in the acetyl-CoA pathway consist of only three folds: the Rossmann fold, TIM barrels, and alpha-beta plaits, while the other half is made up of the remaining 24 folds. The three most common folds are repeatedly recruited and fundamental in the early evolution of enzymatic metabolism, with Rossmann folds and TIM barrels undergoing a rapid early diversification period. Among the 27 different folds employed in the acetyl-CoA pathway, over half are weakly diversified folds that evolved once in the history of life, at the origin of biological CO_2_ fixation. In terms of catalytic strategies, the acetyl-CoA pathway heavily relies on metals and coenzymes, a putative remnant of its geochemical roots. However, amino acids also participate in catalysis by electrostatic stabilization, general acid/base catalysis, metal binding, activation and steric effects.

According to chemolithoautotrophic theories, the origin of metabolism was non-enzymatic, consisting of uncatalyzed and metal-catalyzed reactions (Eakin 1963, Degani and Halmann 1967, Wächtershäuser 1988, Keller et al. 2014, Varma et al. 2018, Muchowska et al. 2019, Preiner et al. 2020, Yi et al. 2022, Henriques Pereira et al. 2022, Beyazay et al. 2023a, Beyazay et al. 2023b, Dherbassy et al. 2023, Song et al. 2024, Kaur et al. 2024, Brabender et al. 2024, Mrnjavac et al. 2025b, Zimmermann et al. 2025, Henriques Pereira et al. 2025, Schlikker et al. 2025). Enzymes may have been preceded by non-encoded catalytic peptides, perhaps made up of a limited amino acid repertoire, that became capable of transition metal binding and folding (Hlouchová 2024). The first enzymes arose sometime between the origin of life and LUCA, given that LUCA already possessed the genetic code and an enzymatic repertoire (Weiss et al. 2016, Moody et al. 2024). Some of the most ancient encoded proteins were likely involved in code expression: ribosomal proteins and translation factors, aminoacyl-tRNA synthetases, polymerases. Enzymes that catalyze the biosynthesis of genetic code components that might have been challenging to synthesize in prebiotic conditions, such as nucleotide synthesis, would closely follow (Mrnjavac et al. 2025b). At this point, a hybrid metabolism of enzymes and metal catalysts could have been in place, and some reactions promoted by metals could start being replaced by enzymes, often by incorporating the metal into the protein structure. Amino acids of early enzymes were carrying out diverse roles in catalysis, with the most common being electrostatic stabilization and general acid/base catalysis. Enzymes replacing metals to promote reactions not only increased rate, but also conferred specificity and brought interconnected reactions to compatible and comparable rates (Wolfenden 2011). This was enabled by the early diversification of folds such as Rossmann and TIM barrels, as well as the singular emergence of specialized, weakly diversified folds. Where solid surfaces were necessary to drive prebiotic reactions, enzymes would have conferred solubility and independence from inorganic mineral surfaces, marking the origin of modern enzymatic metabolism.

## Supplementary Material

fiag041_Supplemental_File
